# Outbreak of *Vibrio cholerae* O1^††^, Mayotte, France, April to July 2024

**DOI:** 10.2807/1560-7917.ES.2024.29.35.2400518

**Published:** 2024-08-29

**Authors:** Sara Mazzilli, Hassani Youssouf, Julie Durand, Marion Soler, Tanguy Cholin, François Herry, Louis Collet, Maxime Jean, Maxime Ransay-Colle, Thierry Benoit-Cattin, Caroline Rouard, Julie Figoni, Harold Noël, Renaud Piarroux, Annabelle Lapostolle

**Affiliations:** 1 ECDC Fellowship Programme, Field Epidemiology path (EPIET), European Centre for Disease Prevention and Control (ECDC), Stockholm, Sweden; 2 Santé publique France (French National Public Health Agency), Saint-Maurice, France; 3 Santé publique France, Cellule régionale d’épidémiologie Mayotte, Mamoudzou, France; 4 Agence Régionale de Santé de Mayotte, Mamoudzou, France; 5 Medical Biology Laboratory, Centre Hospitalier de Mayotte, Mamoudzou, France; 6 Institut Pasteur, Université Paris Cité, Unité des Bactéries pathogènes entériques, Centre National de Référence des Vibrions et du choléra, Paris, France; 7 Inserm, Institut Pierre-Louis d’Épidémiologie et de Santé Publique (IPLESP), Sorbonne Université, Paris, France

**Keywords:** *Vibrio cholerae*, disease outbreaks, drinking water, Mayotte, France

## Abstract

On 22 April 2024, a locally-acquired case of cholera was confirmed in Mayotte. Subsequently, local transmission resulted in eight outbreak clusters with 221 notified cases in densely populated neighbourhoods with limited or no access to drinking water. The last case was detected on 12 July. A case-area targeted intervention strategy was applied to contain the outbreak. However, improving access to drinking water and basic sanitation is crucial to prevent further exposure.

Cholera is an acute bacterial gastrointestinal infection caused by ingestion of the bacterium *Vibrio cholerae*. The bacterium is most commonly transmitted via contaminated food or water [1]†. We describe a cholera outbreak and control measures taken in the French overseas region and department of Mayotte (population approximately 321,000), an archipelago in the Indian Ocean, between Madagascar and the coast of Mozambique.

## Outbreak description

In early February 2024, Comoros declared a cholera epidemic, with Anjouan, the island closest to Mayotte, reporting the highest number of cases. By 27 July 2024, the date of the last recorded case, a total of 10,193 cases have been reported with a fatality ratio of 1.5% [2].

On 18 March 2024, the clinical laboratory at Mayotte hospital (CHM) confirmed the first cholera case imported from Comoros. On 22 April, the first locally-acquired case of cholera was laboratory-confirmed, rapidly followed by local transmission and several cholera outbreak clusters.

The case classification criteria and case definition for the outbreak are presented in the Box.

Box **Criteria for classification of cholera cases and case definition, cholera outbreak, Mayotte, France, April–July 2024**

**Case classification:**

• ***Clinical criteria:***

o Any person with acute watery diarrhoea, vomiting or dying from acute watery diarrhoea.

• ***Laboratory criteria:***

o Detection and identification of *Vibrio cholerae* from a clinical specimen by culture or PCR AND

o Detection of O1 or O139 antigen in the *V. cholerae* isolate by seroagglutination or PCR AND

o Confirmation of toxigenicity by the detection of the *ctxA* gene.

• ***Epidemiological criteria:***

o At least one of the following: exposure to a common source, human-to-human transmission, exposure to contaminated food or drinking water, environmental exposure or travel history from epidemic areas.

**Case definition:**

• ***Probable case:***

o Any person meeting the clinical criteria with an epidemiological link.

• ***Confirmed case:***

o Any person from whom *V. cholerae* O1 has been isolated from a clinical specimen AND meeting the epidemiological criteria OR any person meeting the laboratory criteria.

**Figure 1:**
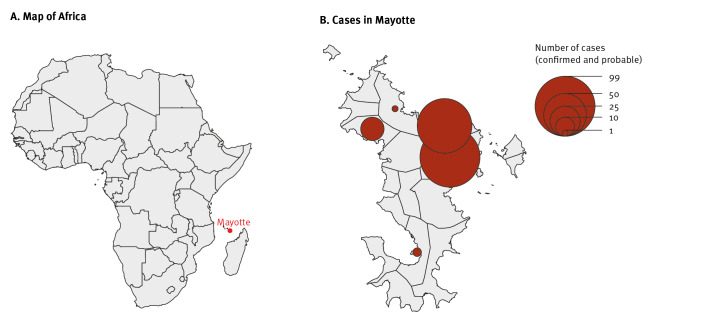
Geographical distribution of non-imported cholera cases, by municipality, Mayotte, France, April–July 2024 (n = 199)

**Figure 2:**
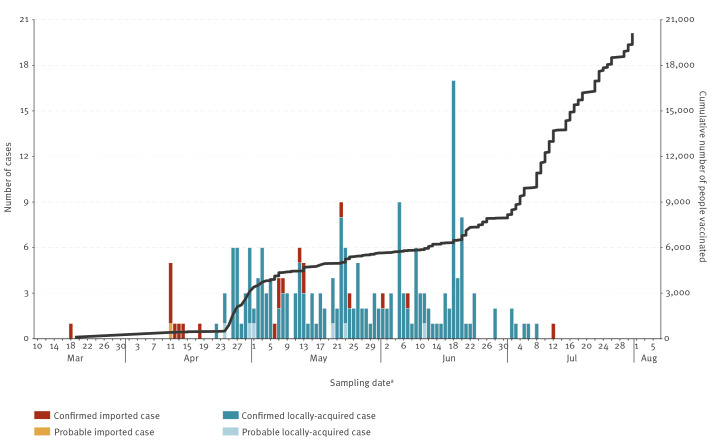
Number of cholera cases (n = 221) and people vaccinated against cholera (n = 20,212), by date, Mayotte, France, April–July 2024

^a^ For probable cases, date of death is presented instead of sampling date.

The last reported case in Mayotte was detected on 12 July. During the epidemic period, 214 confirmed and seven probable cholera cases have been reported, including 22 imported cases (Figure 1, Figure 2). The median age of all cases was 19 years (interquartile range (IQR): 8–33 years), and 123 (56%) cases were male. Fourteen (6%) cases were admitted to intensive care unit. Seven (3%) cases died before being hospitalised: of those, four were confirmed and three were probable cases.

Number of cholera cases per day is presented in the histogram and cumulative number of people vaccinated in the line graph.

The first locally-acquired cholera case was identified in the Kirsoni neighbourhood of Koungou, located in the north-eastern part of the island. Kirsoni is an informal settlement with most people originally from Comoros. One person with a close link to a previously confirmed case died after a sudden onset of gastrointestinal symptoms on 24 April, but no samples were taken before the burial. Several people from the Kirsoni neighbourhood attended the funeral on 25 April. Burial practices may have contributed to the further spread of bacteria in the neighbourhood. Between 22 April and 18 May, 58 cases were detected in Kirsoni; eight were admitted to intensive care and one more person died at home.

**Figure 3:**
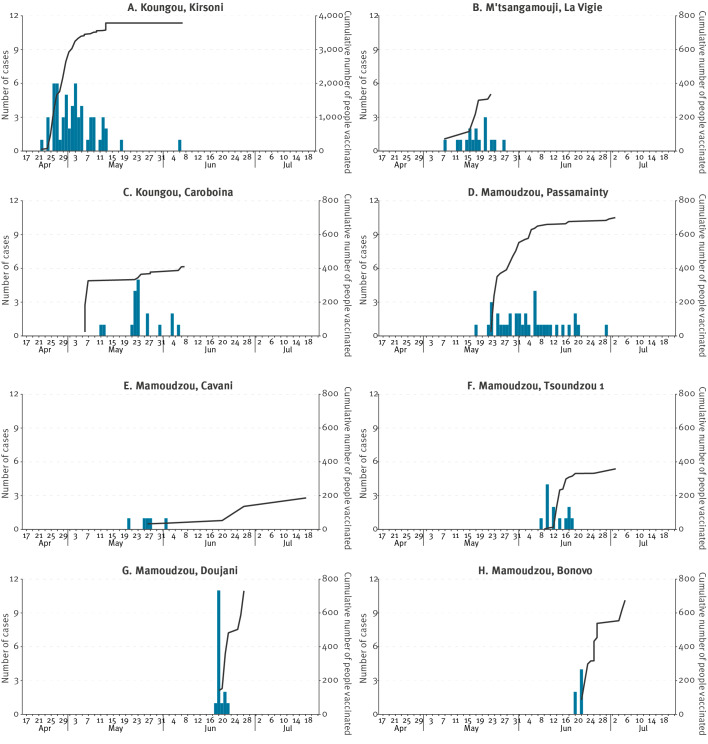
Number of cholera cases (n = 168) and cumulative number of people vaccinated against cholera (n = 7,230), by date and cluster, Mayotte, France, April–July 2024

Seven more clusters of community transmission have been reported on the island up to the end of July (Figure 3, Table).

Numbers of cholera cases per day are presented in the histogram and the cumulative number of people vaccinated in the line graph.

In Panel A., the Y axis representing the cumulative number of vaccinations has a different scale compared with the graphs from the other clusters.

**Table:** Demographic characteristics of cholera cases and prevention measures implemented in outbreak clusters, Mayotte, France, April–July 2024 (n = 221)

Village and municipality of cluster	Date of the first case identified	Number of cases	Sex		Age (years)		Vaccinated with at least one dose of cholera vaccine		Doxycycline prophylaxis for contacts of cases	Contact cases who received doxycycline prophylaxis and Dukoral vaccine
			Male	Female	Median	IQR	Dukoral	Vaxchora		
Koungou, Kirsoni	22 Apr	59	36	23	15	6–30	759	3,045	383	382
M'tsangamouji, La Vigie	7 May	16	14	2	18	14–26	63	270	61	61
Koungou, Caroboina	11 May	18	8	10	11	7–33	98	316	72	69
Mamoudzou, Passamainty	17 May	36	15	21	18	5–34	177	538	162	131
Mamoudzou, Cavani	20 May	5	2	3	22	15–28	53	141	38	37
Mamoudzou, Tsoundzou 1	8 Jun	12	6	6	17	5–34	91	278	53	53
Mamoudzou, Doujani	17 Jun	16	9	7	19	8–24	98	631	51	51
Mamoudzou, Bonovo	18 Jun	6	3	3	11	7–25	128	544	68	67
Not associated to clusters	NA	53^a^	30	22	27	17–43	NA			

IQR: interquartile range; NA: not available.

^a^ Information on sex and age is missing for one person.

For 163 locally-acquired cases, information on water consumption and use were available. Of these, 98 (60%) reported using river water for everyday activities such as washing clothes and the dishes and for personal hygiene, while 30 (18%) also used the river water for drinking.

## Diagnostic strategy

Every person meeting the clinical criteria for cholera underwent a rapid diagnostic test (RDT) (Bioline Cholera Ag O1/O139, Abbott, Lake County, the Unites States (US) or Crystal VC, Arkray, Kyoto, Japan). The RDTs were available at healthcare centres and at CHM. At the same time, the QIAstat-Dx Gastrointestinal Panel 2 with the QIAstat-Dx Analyzer 2.0 (Qiagen, Hilden, Germany) for *V. cholerae* species identification was performed at the CHM. The PCR-positive results were confirmed by culture on selective media, either Thiosulfate-citrate-bile salts-sucrose (TCBS) (Thermo Fisher Scientific, Waltham, US) or chromID Vibrio, (bioMérieux, Marcy l’Étoile, France) followed by identification of *V. cholerae* species by MALDI-TOF (Bruker, Billerica, US). The O1 serogroup determination was performed by slide agglutination (Bio-Rad, Hercules, US) from culture on non-selective media. In case of a negative culture, specific PCRs were performed to detect the presence of *ompW* gene (identification of *V. cholerae* species), *rfbO1* and *rfbO139* genes encoding the O1 and O139 antigen and *hlyA* gene to distinguish a possible vaccine strain (with truncated *hlyA* gene) from the wild strain (with complete *hlyA* gene) [3].

## Laboratory findings

At the beginning of the outbreak, all isolates were confirmed at the French National Reference Center for Vibrios and cholera (NRC) at the Institut Pasteur in Paris. Later, only a subset of isolates were confirmed, as recommended by the World Health Organization (WHO): two or three isolates per cluster per week from locally-acquired cases and imported cases. As of 12 July, 126 isolates among the 221 cases were confirmed at the NRC with *ctxA* gene identification [4,5], serotype determination (slide agglutination with Ogawa and Inaba antisera) and whole genome sequencing [6]. All isolates in Mayotte were confirmed as *V. cholerae* O1 Ogawa El Tor of the seventh pandemic lineage (7PET) carrying an extended-spectrum beta-lactamase (PER-7), resistance to azithromycin and ciprofloxacin, but sensitive to doxycycline.

## Prevention and control measures

### Early case detection and enhanced surveillance

Systematic PCR screening for travellers from East Africa and active case finding for travellers from Comoros have been implemented at entry points to Mayotte.

In April 2024, a training campaign was conducted for healthcare personnel in Mayotte in preparation for the detection of cholera cases. Rapid diagnostic tests were distributed to the four principal primary healthcare centres and the hospital of Mayotte, and early warning systems were implemented by health staff to enable alerting the regional health agency by phone to respond promptly to positive RDT/PCR results.

Hospital surveillance was further strengthened through the bi-daily transmission of laboratory results of multiplex PCR tests to regional health authorities.

Mayotte's public health surveillance system included community-based surveillance (CBS), which was implemented by non-governmental organisations (NGOs) in the most vulnerable neighbourhoods and coordinated by the regional office of the National Institute of Health, the regional health agency and the emergency medical assistance service. The NGOs were trained to actively search cholera cases and raise community awareness. They participated in the early warning system by alerting health authorities if they identified suspected cases of cholera.

### Case-area targeted interventions

In this outbreak, a case-area targeted intervention (CATI) strategy was applied to prevent spatial propagation by containing clusters, as previously described and implemented in other settings [7-10]. The response focused on reinforcing household-level sanitation and hygiene measures with distribution of hygiene kits, including soap, hydroalcoholic solution, closed buckets and chlorine tablets.

The administration of antibiotic chemoprophylaxis, using single-dose doxycycline (2–4 mg/kg for children aged < 12 years and 300 mg for others) for household members and close contacts of confirmed cholera patients, was also implemented.

Oral cholera vaccination was offered to people living within 50 m of a confirmed case, or mass vaccination was performed in the neighbourhood where a cluster had been identified. People were vaccinated with Vaxchora (Emergent BioSolutions, Rockville, US) except children aged < 2 years, pregnant people, immunocompromised persons and persons treated with chemoprophylaxis, who received Dukoral (Valneva, Saint-Herblain, France).

Active case finding and educational campaigns on health promotion and cholera prevention measures were also implemented for neighbourhoods affected by epidemic clusters.

A temporary vaccination and screening centre was opened in Kirsoni, in addition to a permanent water distribution point.

### Improvement of water supply and mass vaccination

Since June, several drinking water sources have been established in disadvantaged neighbourhoods that had limited or no access to drinking water.

A mass vaccination campaign was launched in July, beginning in the neighbourhoods considered most susceptible to the spread of the epidemic, with the aim of vaccinating the entire at-risk population of the island (Figure 2).

## Discussion

In January 2023, the WHO classified the global resurgence of cholera as a grade 3 emergency, its highest internal level for emergencies [1].The global resurgence of cholera and favourable local conditions allowed for an introduction and spread of cholera in Mayotte. The containment measures implemented successfully prevented the importation of the disease to mainland France.

Mayotte has been a French region and department since 2011. On 1 January 2024, the mean age of Mayotte’s population of 321,000 persons was 23 years, with an average annual population growth of 3.8%. A high birth rate and immigration have contributed to the population growth [11]. The migration flow comes mainly from Comoros [12].

Almost 77% of the population live below the poverty line, and in 2017, only 71% of the population had access to clean water at their home [13]. Due to low rainfall during the 2022–2023 season in Mayotte, water levels in the hill basins were at negative records. As a result, there were regular cuts in water distribution, up to 54 hours (2 of 3 days), that were ongoing during the cholera outbreak [14].

The living conditions of many inhabitants have become more conducive to cholera as climate change, population growth and increasing informal urbanisation have exacerbated the need for improvement in water resources and wastewater management infrastructure in Mayotte [15].

Our results indicate that densely populated neighbourhoods with limited or no access to drinking water and intersected by easily contaminated surface water sources like rivers, were disproportionately affected by the cholera epidemic. More than half of the cases with information available on water consumption, reported using river water for everyday activities.

The resurgence of cholera cases on the African continent and in Comoros has steadily spilled over, leading to the introduction of the pathogen in the informal neighbourhoods of Mayotte. Residents in these neighbourhoods often have a history of undocumented immigration and thus they may not seek medical care due to their undocumented residence status [16]. This may contribute to cases not being diagnosed or being diagnosed late and hindering the implementation of necessary containment methods.

As part of the response effort, regional authorities implemented CATI, which included mass oral cholera vaccination, primarily using Vaxchora, in neighbourhoods with epidemic clusters. Since July, a mass vaccination campaign was launched. In a clinical study, Vaxchora was 90% effective 10 days after vaccination and 80% effective 3 months after [17]. The limited evidence on the effectiveness of this vaccine in containing an outbreak [18,19] highlights the need for an evaluation of this intervention as a control measure. Moreover, its long-term effectiveness in preventing future cholera outbreaks remains unclear. Dukoral has been administered to children < 2 years; however, its protective effectiveness in this age group has not been confirmed.

Given the continuous arrival of people from neighbouring countries experiencing ongoing cholera outbreaks, the risk of reintroduction of the pathogen endures. It is crucial to improve access to safe drinking water, ensure household water supplies and provide basic sanitation in informal settlements to prevent durably cholera and other waterborne diseases in Mayotte [8]. Notably, regions with access to safe drinking water and sanitation have a significantly reduced risk of local cholera transmission [20].

## Conclusion

The cholera epidemic in Mayotte underscores the success of containment efforts through the case-area targeted intervention strategy, which aims to prevent spatial spread by containing clusters and includes raising awareness among at-risk populations, vaccination and effective case management. These measures must be part of a multi-sectoral response. Ensuring access to clean water and sanitation for all inhabitants is crucial not only for the long-term control and elimination of cholera but also for addressing health inequalities, enhancing preparedness, and safeguarding public health. Our findings should contribute to the ongoing discourse on effective strategies for mitigating and controlling the current global cholera resurgence.

## ^†^Erratum

The references were incorrectly numbered at the time of publication. This mistake was corrected on 30 August 2024.

## ^††^Author’s correction

The serogroup was missing from the title at the time of publication. This was corrected on 10 September 2024 at the request of the authors.
