# Erratum for Euro Surveill. 2024;29(35)

**DOI:** 10.2807/1560-7917.ES.2024.29.36.240830e

**Published:** 2024-09-05

**Authors:** Eurosurveillance editorial team

**Affiliations:** 1 European Centre for Disease Prevention and Control (ECDC), Stockholm, Sweden

In the article entitled ‘Outbreak of *Vibrio cholerae*, Mayotte, France, April to July 2024’ by Mazzilli et al., published on 29 August 2024, the references were incorrectly numbered at the time of publication. This mistake was corrected on 30 August 2024.

